# Editorial: Cross-species transmission of viral infections

**DOI:** 10.3389/fmicb.2026.1955124

**Published:** 2026-08-20

**Authors:** Alexandro Guterres

**Affiliations:** Department of Genetics, Ribeirão Preto Medical School, University of São Paulo—USP, São Paulo, Brazil

**Keywords:** cross-species transmission, genetic recombination, genomic surveillance, molecular evolution, One Health, pathogen emergence, viral dynamics, zoonotic spillover

The emergence and re-emergence of zoonotic diseases represent a formidable and often unpredictable threat to global public health, as demonstrated by the devastating impact of recent viral outbreaks. These events are increasingly driven by a complex interplay of anthropogenic activities, human-animal coexistence, and enhanced global connectivity, which provide unprecedented opportunities for pathogen spillover and genetic recombination. Understanding the intricate mechanisms of viral dynamics at the human-animal-environment interface requires a multidisciplinary “One Health” approach that integrates robust genomic surveillance with spatial and epidemiological data. This Research Topic brings together nine high-impact studies that utilize advanced molecular and metagenomic tools to decipher the evolutionary trajectories of high-risk pathogens, ranging from well-established threats like Middle East Respiratory Syndrome Coronavirus (MERS-CoV) and Influenza A to underappreciated viruses in wildlife and domestic reservoirs. By mapping transmission networks and identifying novel genetic variants, these contributions provide a proactive scientific foundation for pandemic preparedness and the development of effective biosecurity policies (Figure 1).

**Figure 1:**
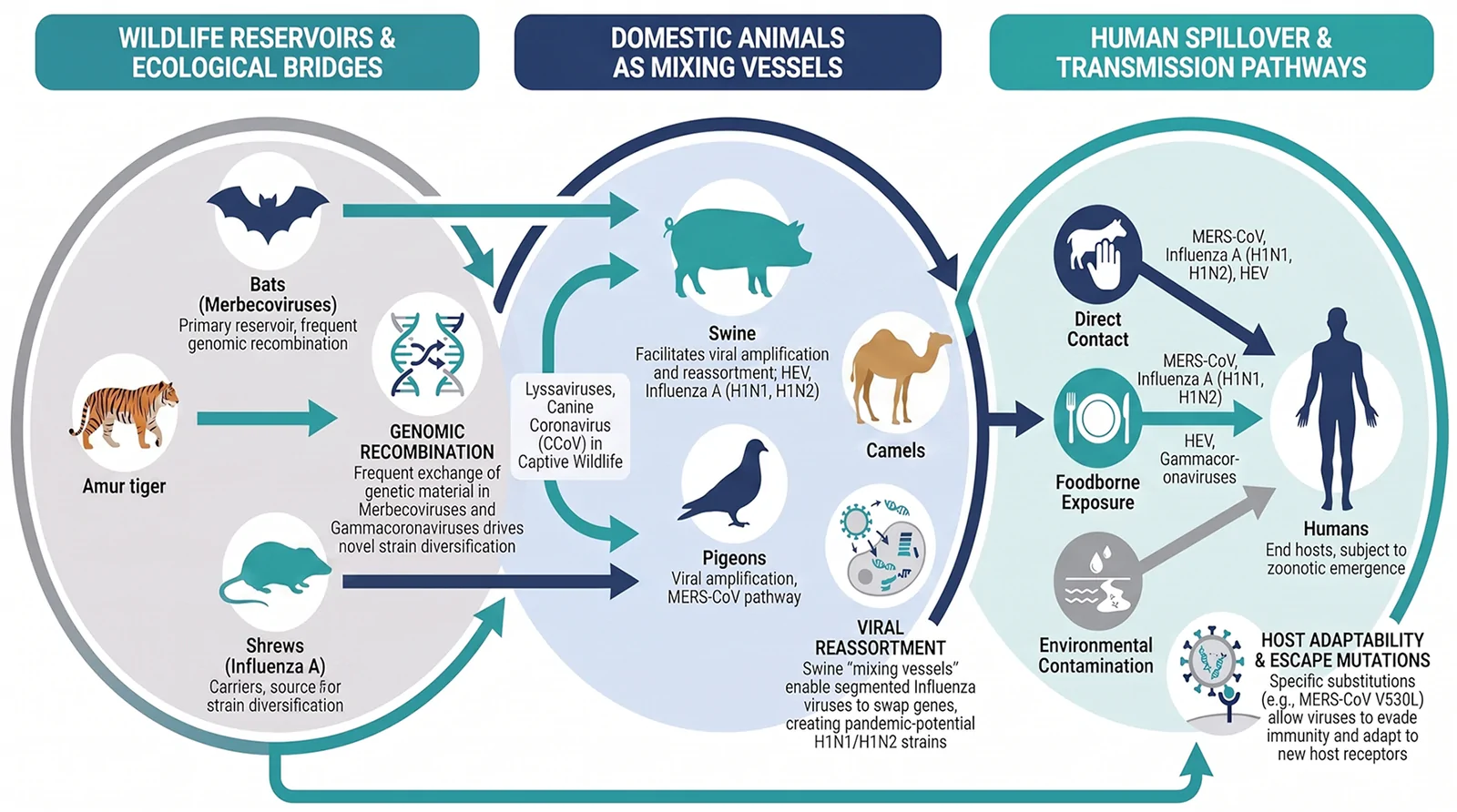
The One Health framework for proactive genomic surveillance and viral dynamics. The diagram illustrates the interconnectedness of wildlife (e.g., Amur tigers, Asian house shrews, and bats), domestic animals (e.g., swine and pigeons), and humans in the transmission and evolution of viral pathogens. Key evolutionary drivers, such as genomic recombination and reassortment, are highlighted as mechanisms for host-jumping and therapeutic evasion. Arrows represent documented or potential viral spillover pathways and transmission networks identified by the contributing studies in this Research Topic.

## Molecular dynamics and immune evasion in coronaviruses

Coronaviruses continue to be a primary focal point of genomic surveillance due to their broad host range and high propensity for genomic recombination and point mutations. Han et al. provide a critical “cross-species transmission alert” by documenting a novel recombinant coronavirus, TigerCoV-JL1, infecting a captive Amur tiger in China (Figure 1). Utilizing metagenomic next-generation sequencing, the researchers identified that this strain represents a unique canine-raccoon dog recombinant, carrying genetic signals from domestic canine coronaviruses and raccoon dog lineages within the ORF1a and Spike (S) genes. This discovery highlights the susceptibility of endangered felids to viral jumps and underscores the risks inherent in semi-captive environments where high-density interfaces between carnivores facilitate complex evolutionary pathways. In parallel, the study by Pan et al. delves into the molecular determinants of immune evasion within the merbecovirus subgenus. By evaluating 10 anti-MERS-CoV monoclonal antibodies (mAbs) against a diverse panel of pseudoviruses, they demonstrated how the V530L substitution in the MERS-CoV clade B, identified during the 2015 South Korean outbreak, drastically reduces the neutralization potency of the potent mAb m336 (Pan et al.). Advanced structural modeling using AlphaFold3 revealed that this substitution induces significant steric changes in the receptor-binding domain (RBD), disrupting high-affinity hydrogen bonds between the antibody and the viral protein. These findings collectively emphasize that while large-scale recombination events expand host range, discrete molecular adaptations can compromise therapeutic efficacy, necessitating the development of multi-epitope strategies for future pandemic preparedness (Pan et al.; Han et al.).

## Domestic pigs as “mixing vessels” for viral reassortment and recombination

The role of domestic animals as “mixing vessels” for viral evolution remains a critical pillar of zoonotic risk assessment (Figure 1). Pigs, in particular, possess receptors for both avian and human influenza viruses, facilitating complex genetic reassortment, and the emergence of strains with pandemic potential. Cavicchio et al. exemplify the importance of sustained monitoring through a decade-long passive surveillance study (2013–2022) in Northern Italy, which identified 12 distinct Swine Influenza A (swIAV) genotypes, including the first European reports of the Novel 1 and Novel 2 genotypes. Their phylogenetic analysis revealed that genetic clustering is strongly influenced by anthropogenic factors, such as farm ownership and management practices, with documented instances of human-to-swine transmission sustaining local viral circulation and increasing the complexity of the internal gene constellations. Parallel surveillance in China by Zhao et al. characterized a novel triple-reassortant H1N2 virus, designated as genotype G21, which acquired its HA gene from Eurasian avian-like H1N1 and its internal genes from the 2009 pandemic H1N1. Molecular analysis of this isolate identified the N31 substitution in the M2 protein, conferring adamantane resistance, and the S42 mutation in the NS1 protein, which is known to influence virulence in mammalian models. Furthermore, experimental infection in BALB/c mice confirmed moderate pathogenicity and efficient replication in the lungs and nasal turbinates, signaling its potential for further regional adaptation. Beyond respiratory threats, the study by Liu et al. underscores the significant food safety implications of the human-swine interface across Asia. By analyzing 348 complete genomes of Hepatitis E virus (HEV), they identified 34 recombination events, predominantly localized within the RdRp region, including 14 cross-species exchanges between swine and human strains (Liu et al.). The high frequency of HEV-4 recombination in China appears to be driven by the intensive nature of the swine industry and high-density interaction hubs, reinforcing the need for stricter quarantine and molecular monitoring across the entire food production chain to mitigate the risk of zoonotic foodborne transmission (Liu et al.; Zhao et al.).

## Wildlife reservoirs and the characterization of novel avian pathogens

Wildlife reservoirs serve as the primary origin of future pandemics, yet their internal viral landscapes often remain opaque. Huangfu et al. address this gap through an extensive metagenomic survey of the Asian house shrew (*Suncus murinus*) in tropical Hainan, China. By performing RNA sequencing on multiple tissues, the researchers identified a staggering 192 RNA viruses belonging to 32 known families, including significant human pathogens such as Henipavirus, Langat virus (LGTV), Amur virus (AMRV), and Influenza A (H1N1) (Huangfu et al.). Their findings challenge the traditional perception of shrews as low-diversity reservoirs and identify them as a critical “ecological bridge” facilitating viral sharing between otherwise distantly related hosts, such as rodents and bats (Figure 1). This diversity is likely enhanced by the tropical ecosystem of Hainan, which supports stable host and vector populations year-round. In the avian sector, Tan et al.'s provide critical insights into the taxonomy and distribution of pigeon gammacoronavirus (PgCoV). Utilizing high-throughput sequencing, they assembled a complete 27,538 nt genome, identifying unique accessory protein profiles, including a novel ORFx2 and the absence of proteins 3a and 3b (Tan et al.). Comparative analysis of five conserved domains (including 3CLpro and RdRp) revealed amino acid differences exceeding 8.3% compared to known species, providing robust evidence for classifying PgCoV as a novel species within the genus *Gammacoronavirus*. Furthermore, their epidemiological survey documented a remarkably high flock-level prevalence of 97.73%, with RT-PCR evidence of viral replication in intestinal and kidney tissues, signaling the virus's potential pathogenicity in the digestive and urinary systems of pigeons. Collectively, these studies underscore the necessity of integrating understudied synanthropic and avian species into global virological surveillance to monitor emerging biosecurity threats (Tan et al.; Huangfu et al.).

## Long-term surveillance and human-centric transmission networks

The complexity of viral dynamics is further elucidated by combining long-term field surveillance with human-centric molecular network analyses. Weinberg et al. provide a significant regional perspective from Israel, where 27 years of surveillance (1995–2022) encompassing over 42,000 animals demonstrated a minimal risk of *Rabies lyssavirus* spillover from bats to terrestrial mammals (Weinberg et al.). Despite the presence of 32 bat species, including the synanthropic Egyptian fruit bat which frequently forages in urban environments, zero positive cases were identified among the 294 bats tested using the historical gold-standard TAF method. This absence of infection reinforces the epidemiological distinction between Old and New World bat populations and highlights that reservoir roles are often region-specific, suggesting that bats in this region remain largely isolated from terrestrial *Lyssavirus* transmission cycles. In contrast, in the context of direct human-to-human transmission, Wu et al. utilize molecular transmission networks to track HIV-1 among men who have sex with men (MSM) in Ningxia, China. Their analysis of 296 *pol* gene sequences revealed an overall clustering rate of 43.6%, with the CRF07_BC subtype (57.1%) emerging as a dominant driver of localized spread (Wu et al.). Notably, the study identified an alarming 38.2% prevalence of acquired drug resistance among sequenced individuals, demanding targeted public health interventions based on urban spatial connectivity and a “network-based approach” to effectively manage the spread of the epidemic. These contrasting studies emphasize that while some natural reservoirs pose low immediate risks, human social networks remain high-velocity conduits for viral dissemination and evolution (Weinberg et al.; Wu et al.).

In conclusion, the studies presented in this Research Topic reaffirm that genomic surveillance is a proactive sentinel for global health security rather than merely a descriptive tool. By integrating data across the human-animal-environment interface, researchers have mapped recombination hotspots in Hepatitis E and swIAV, identified cross-species transmission potential in felid coronaviruses, and defined novel species of avian gammacoronaviruses. These contributions provide a robust scientific foundation for evidence-based biosecurity and public health policies, highlighting that discrete molecular adaptations can significantly compromise therapeutic efficacy or alter pathogenicity. The adoption of a multidisciplinary “One Health” framework is essential to stay ahead of viral adaptation, particularly as anthropogenic activities and ecological shifts continue to blur the boundaries between domestic and wild habitats. Ultimately, this Research Topic underscores that protecting global biodiversity, ensuring food safety, and monitoring high-risk human networks are intrinsically linked strategies for the mitigation of future pandemic threats in an ever-changing world.

